# Diagnostic performance of two-dimensional shear wave elastography and attenuation imaging for fibrosis and steatosis assessment in chronic liver disease

**DOI:** 10.1007/s10396-024-01473-5

**Published:** 2024-06-29

**Authors:** Tamaki Kobayashi, Takuma Nakatsuka, Masaya Sato, Yoko Soroida, Hiromi Hikita, Hiroaki Gotoh, Tomomi Iwai, Ryosuke Tateishi, Makoto Kurano, Mitsuhiro Fujishiro

**Affiliations:** 1 Department of Clinical Laboratory Medicine, The University of Tokyo, Tokyo, 113-8655 Japan; 2 Department of Gastroenterology, Graduate School of Medicine, The University of Tokyo, 7-3-1 Hongo, Bunkyo-Ku, Tokyo, 113-8655 Japan

**Keywords:** Shear wave elastography, Attenuation imaging, Liver fibrosis, Hepatic steatosis

## Abstract

**Purpose:**

We investigated the diagnostic performance of two-dimensional shear wave elastography (2D-SWE) and attenuation imaging (ATI) in detecting fibrosis and steatosis in patients with chronic liver disease (CLD), comparing them with established methods.

**Methods:**

In 190 patients with CLD, 2D-SWE and vibration-controlled transient elastography (VCTE) were used for liver stiffness measurement (LSM), and ATI and controlled attenuation parameter (CAP) were used for steatosis quantification. The correlations between these new and established methods were analyzed.

**Results:**

Significant correlations were found between 2D-SWE and VCTE (r = 0.78, *P* < 0.001), and between ATI and CAP (r = 0.70, *P* < 0.001). Liver stiffness tended to be lower with 2D-SWE compared with that with VCTE, especially in cases with higher LSM, and ATI was less influenced by skin-capsular distance than CAP. Area under the receiver-operating characteristics curves (AUCs) and optimal cut-offs of 2D-SWE for diagnosing liver fibrosis stages F2, F3, and F4 were 0.73 (8.7 kPa), 0.79 (9.1 kPa), and 0.88 (11.6 kPa), respectively. The AUCs and optimal cut-offs of ATI for diagnosing hepatic steatosis grades S1, S2, and S3 were 0.91 (0.66 dB/cm/MHz), 0.80 (0.79 dB/cm/MHz), and 0.88 (0.86 dB/cm/MHz), respectively. A subgroup analysis of 86 patients with metabolic dysfunction-associated steatotic liver disease also demonstrated good performance for 2D-SWE and ATI.

**Conclusion:**

2D-SWE and ATI performed comparably with conventional VCTE and CAP in evaluating CLD, offering reliable alternatives for diagnosing liver fibrosis and steatosis.

**Supplementary Information:**

The online version contains supplementary material available at 10.1007/s10396-024-01473-5.

## Introduction

Chronic liver disease (CLD) accounts for a significant burden of disease and costs worldwide, and its precise and appropriate management is an urgent issue [1]. The prognosis and management of CLD are largely dependent on the extent and progression of liver fibrosis. Liver biopsy remains the gold standard for staging liver fibrosis; however, it has several limitations. Sampling bias and intra- and inter-observer variability can lead to over- and under-diagnosis of fibrosis, respectively [2]. Furthermore, liver biopsy is an invasive test with a high cost and rare but life-threatening complications [3]. For these reasons, it is not practical to perform liver biopsies in many patients with CLD solely for fibrosis staging.

Noninvasive diagnostic methods (e.g., elastography) to measure liver stiffness, which corresponds to the physical properties of the liver parenchyma, have been developed and are widely used for liver fibrosis. Vibration-controlled transient elastography (VCTE) using FibroScan® (Echosens, Paris, France) [4] has high diagnostic performance for liver fibrosis in CLD of various etiology [5] and is referenced in many international guidelines [6, 7]. More recently, ultrasound-based elastography techniques, such as point shear wave elastography (pSWE) and two-dimensional SWE (2D-SWE), have been developed [8, 9].

Non-alcoholic fatty liver disease (NAFLD) has become the most common CLD worldwide, and is the leading cause of liver-related morbidity and mortality [10]; therefore, hepatic steatosis assessment has great clinical importance. Furthermore, because of its strong epidemiological and pathogenic relationship with metabolic dysfunction and insulin resistance, NAFLD has recently been renamed metabolic dysfunction-associated steatotic liver disease (MASLD) [11, 12]. Several methods based on ultrasound attenuation measurements have been developed to evaluate steatosis. Controlled attenuation parameter (CAP), a VCTE-guided ultrasound attenuation measurement [13], has high diagnostic performance for hepatic steatosis in CLD of various etiologies [14]. In recent years, new methods have emerged for quantifying ultrasound attenuation using B-mode imaging [15–17].

Canon Medical Systems developed a new 2D-SWE method with propagation maps for liver stiffness measurement (LSM) and proprietary attenuation imaging (ATI), as a new ultrasound diagnostic method for hepatic steatosis [18]. Ultrasound-based techniques allow proper region of interest (ROI) selection by referring to B-mode ultrasound images and avoiding interfering structures. Furthermore, ultrasound-based techniques do not require a dedicated probe other than an abdominal ultrasound device, making them more widely available. However, data on 2D-SWE and ATI assessments for liver fibrosis and steatosis are limited. In this study, we investigated the diagnostic performance of 2D-SWE and ATI on the Canon ultrasound system for liver fibrosis and steatosis in patients with CLD of various etiologies; additionally, we analyzed factors affecting the measured values.

## Material and methods

### Patients

We prospectively examined 190 patients with CLD who visited our liver clinic and underwent simultaneous VCTE, CAP, 2D-SWE, and ATI measurements between January 2020 and July 2023. All the patients agreed to participate in the study and provided written informed consent. This study was approved by the University of Tokyo Medical Research Center Ethics Committee (approval number: 2019343NI), and was performed in accordance with the ethical guidelines of the Declaration of Helsinki. All patients with steatotic liver disease were classified into MASLD, MASLD and increased alcohol intake (MetALD), or ALD according to the Delfi criteria [11, 12].

### VCTE and CAP

The FibroScan® Touch 502 (Echosens, Paris, France) was used for the LSM-VCTE and CAP measurements. A probe was placed at the intercostal space along the midaxillary line, aiming at the right lobe of the liver. Ten LSM scans coupled with CAP acquisition were performed for each patient. The LSMs were accepted only when the success rate was ≥ 60%, and the interquartile range (IQR) was < 30% of the median for LSM > 7.1 kPa. VCTE and CAP were performed by an experienced medical sonographer (T.K.) or an experienced hepatologist (T.N.), each of whom has a training certificate for FibroScan® issued by Echosens.

### 2D-SWE and ATI

Examinations were performed using a Canon Aplio a550 or i700 ultrasound system (Canon Medical Systems, Tochigi, Japan). LSM-SWE and ATI were performed with 6-MHz, 8-MHz, or i8CX5 probes using a similar approach at the same intercostal space as VCTE at the location of the liver parenchyma with good B-mode delineation and without vascular structures or artifacts. For 2D-SWE, the measurement box was placed 2 cm below the liver surface, and a circular measurement ROI with a diameter of 1 cm was placed on the liver parenchyma where parallel propagation lines appeared with constant line spacing on the propagation map. The patients were asked to hold their breath lightly for a few seconds, during which six consecutive LSMs were performed. The LSMs were adopted when the IQR was < 30% of the median on the kilopascals display, and the median value was used as the representative value. For ATI, the measurement box was placed at the liver parenchyma, such that the top of the box was positioned at a depth twice the thickness of the subcutis, and five consecutive measurements were performed. 2D-SWE and ATI were performed by an experienced medical sonographer (T.K.) or an experienced hepatologist (T.N.), each of whom has approximately 10 years of ultrasound experience.

### Histopathological evaluation

All liver biopsy specimens were obtained percutaneously from the right lobe of the liver under ultrasound guidance using a 15-gauge needle with a biopsy specimen notch 20 mm in length. Based on a previous report [19] on patients with steatotic liver disease, the fibrosis stage was determined using the Brunt criteria [20]. As for patients with other liver diseases, fibrosis stage was determined using the METAVIR scoring system [21]. Both systems classify the degree of liver fibrosis into five stages: F0 (no fibrosis), F1 (mild fibrosis), F2 (moderate fibrosis), F3 (severe fibrosis), and F4 (cirrhosis). The steatosis grade was determined according to the histological scoring system for NAFLD as follows: S0 (< 5%, none), S1 (5–33%, mild), S2 (> 33–66%, moderate), and S3 (> 66%, severe) [22]. All liver biopsy specimens were diagnosed by one of several experienced pathologists with at least 10 years of experience at our institution and who were blinded to the clinical data.

### Statistical analysis

Patient parameters and laboratory results were summarized using descriptive statistics. Continuous variables were presented as mean and standard deviation or median and IQR. Correlations were analyzed using the Pearson’s product-rate correlation coefficient. Linear regression was performed to assess the correlation between the factors affecting LSM-VCTE, LSM-SWE, CAP, and ATI. A Bland–Altman analysis was performed to assess the concordance between LSM-SWE and LSM-VCTE. A trend test was performed using the Jonckheere-Terpstra test. The area under the curve (AUC) was evaluated to confirm the diagnostic performance for liver fibrosis and steatosis, and the optimal cut-off values were determined using the Youden index. The significance difference of AUCs was evaluated using DeLong’s test. Statistical analysis was performed using the R software package (version 4.3.0; R Development Core Team, Vienna, Austria), and *P* values of < 0.05 were considered significant.

## Results

### Patient characteristics

Patient characteristics are shown in Table 1. The causes of liver disease were hepatitis B in 28 patients, hepatitis C in 39 (all after achieving sustained virological response), MASLD in 86, autoimmune hepatitis in 14, primary biliary cholangitis in 11, alcohol-related liver disease (ALD) in nine, and cryptogenic liver disease in three. Of these, 66 patients underwent liver biopsy (Table S1).

**Table 1:** Patient characteristics

Variable	Patients with CLD (n = 190)	
BMI	26.5	(23.3–30.1)
Age	64	(52–73)
Male, %	97	(51)
Platelet count, × 10^3^/μL	218	(182–261)
Albumin, g/dL	4.3	(4.1–4.5)
AST, U/L	30	(22–50)
ALT, U/L	29	(17–52)
GGT, U/L	40	(22–90)
Total bilirubin, mg/dL	0.8	(0.7–1.1)
FIB-4 index	1.60	(1.17–2.32)
SCD, cm	1.77	(1.44–2.07)
LSM-VCTE, kPa	6.7	(4.0–11.3)
LSM-SWE, kPa	6.9	(5.4–9.0)
CAP, dB/m	262	(216–314)
ATI, dB/cm/MHz	0.69	(0.59–0.81)
Fibrosis stage		
0–1	17	(25.8)
2	14	(21.2)
3	22	(33.3)
4	13	(19.7)
Steatosis grade		
0	14	(21.2)
1	27	(40.9)
2	18	(27.2)
3	7	(10.6)
Etiology		
HBV	28	(14.7)
HCV (SVR)	39	(20.5)
MASLD	86	(45.3)
ALD	9	(4.7)
AIH	14	(7.4)
PBC	11	(5.8)
Cryptogenic	3	(1.6)

Data are expressed as the median (25th–75th percentiles) or number (percentages). CLD: chronic liver disease, BMI: body mass index, AST: asparagine aminotransferase, ALT alanine aminotransferase, GGT: γ-glutamyltransferase, FIB-4: fibrosis 4, SCD: skin-capsular distance, LSM: liver stiffness measurement, VCTE: vibration-controlled transient elastography, SWE: shear wave elastography, CAP: controlled attenuation parameter, ATI: attenuation imaging, HBV: hepatitis B virus, HCV: hepatitis C virus, SVR: sustained virological response, MASLD: metabolic dysfunction-associated steatotic liver disease, ALD: alcohol-related liver disease, AIH: autoimmune hepatitis, PBC: primary biliary cholangitis

### Relationship between 2D-SWE and VCTE and associated factors

**Fig. 1:**
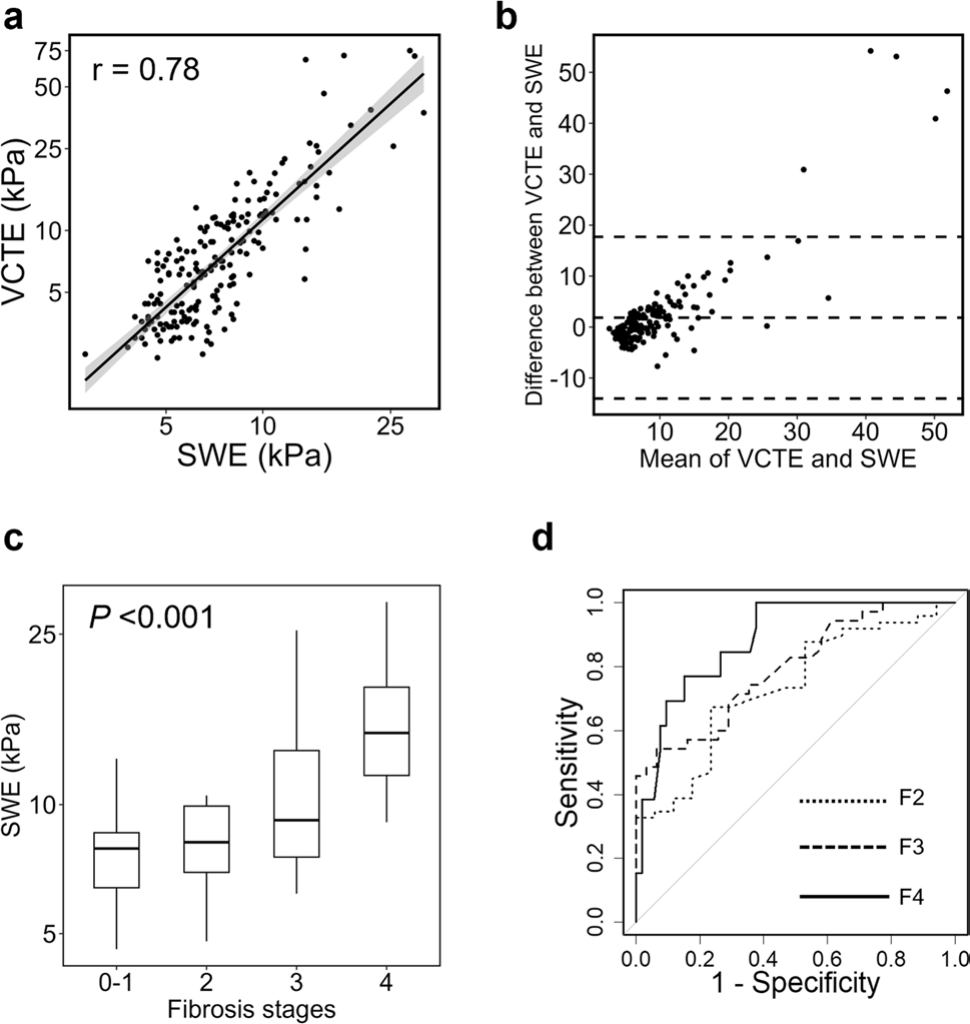
Diagnostic performance of 2D-SWE in patients with chronic liver disease. **a** 2D-SWE and VCTE showed a positive correlation (r = 0.78, *P* < 0.001, Pearson product-rate correlation coefficient analysis). **b** Bland–Altman plots for 2D-SWE and VCTE. The solid line represents the mean of the difference in liver stiffness values, and the dashed lines define the limits of agreement (95% upper and lower limits). **c** LSM using 2D-SWE significantly increased according to the liver fibrosis stage (*P* < 0.001, Jonckheere-Terpstra trend test). **d** Receiver operating characteristic curves of 2D-SWE for diagnosing the stages of liver fibrosis. 2D-SWE: two-dimensional shear-wave elastography, VCTE: vibration-controlled transient elastography, LSM: liver stiffness measurement

A strong positive correlation was observed between LSM using 2D-SWE (LSM-SWE) and LSM using VCTE (LSM-VCTE) (r = 0.78, *P* < 0.001) (Fig. 1a). A Brandt–Altman analysis revealed that the mean difference between LSM-SWE and LSM-VCTE was 1.84 ± 15.87 kPa, and the 95% upper and lower limits of agreement were 17.70 kPa and -13.98 kPa, respectively (Fig. 1b). As shown in the plot, there was a proportional error between LSM-SWE and LSM-VCTE, where the difference in measurements increased as LSM increased.

Univariate analysis revealed that the following factors were significantly associated with LSM-SWE: body mass index (BMI), male sex, platelet count, albumin, asparagine aminotransferase (AST), alanine aminotransferase (ALT), γ-glutamyltransferase (GGT), total bilirubin, and skin-capsular distance (SCD). Factors associated with LSM-VCTE were similar to those associated with LSM-SWE. Multivariate analysis showed that platelet count and GGT level were independently associated with LSM-SWE and LSM-VCTE (Table 2).

**Table 2:** Factors associated with 2D-SWE and VCTE

Variable	2D-SWE				VCTE			
	Univariate		Multivariate		Univariate		Multivariate	
	β	*P* value	β	*P* value	Β	*P* value	Β	*P* value
BMI	0.31	** < 0.001**	0.22	0.10	0.41	** < 0.001**	0.32	**0.02**
Age	– 0.03	0.70			0.02	0.80		
Male	0.20	**0.01**	– 0.04	0.70	0.16	**0.03**	– 0.05	0.50
Platelet count	– 0.17	**0.02**	– 0.21	**0.01**	– 0.22	**0.002**	– 0.28	** < 0.001**
Albumin	– 0.21	**0.01**	– 0.08	0.30	– 0.24	**0.001**	– 0.10	0.20
AST	0.39	** < 0.001**			0.39	** < 0.001**		
ALT	0.27	** < 0.001**	0.06	0.40	0.31	** < 0.001**	0.09	0.20
GGT	0.41	** < 0.001**	0.24	** < 0.001**	0.38	** < 0.001**	0.22	**0.002**
Total bilirubin	0.23	**0.002**	0.15	0.06	0.19	**0.01**	0.11	0.14
SCD	0.33	** < 0.001**	0.08	0.60	0.39	** < 0.001**	0.13	0.30

*SWE* shear wave elastography, *VCTE* vibration-controlled transient elastography, *BMI* body mass index, *AST* asparagine aminotransferase, *ALT* alanine aminotransferase, *GGT* γ-glutamyltransferase, *SCD* skin-capsular distance

LSM-SWE was performed using two different ultrasound devices from Canon (Aplio a550 and Aplio i700). There was no significant difference in the patient backgrounds between the groups measured with the Aplio a550 and Aplio i700, and LSM-SWE results obtained with the Aplio a550 and Aplio i700 were comparable (Table S2). Furthermore, the correlation between LSM-SWE and LSM-VCTE was equally strong whether using the Aplio a550 (r = 0.73, *P* < 0.001) or the Aplio i700 (r = 0.79, *P* < 0.001) (Fig. S1).

### Diagnostic ability of LSM-SWE for liver fibrosis staging

In cases where a liver biopsy was performed, LSM-SWE measurements increased significantly according to the liver fibrosis stage (Fig. 1c). The AUCs of LSM-SWE for diagnosing fibrosis stages F2, F3, and F4 were 0.73, 0.79, and 0.88, respectively (Fig. 1d). Optimal cut-off levels of LSM-SWE for diagnosing liver fibrosis were 8.7 kPa for F2 (sensitivity [se]: 67.3%, specificity [sp]: 76.5%), 9.1 kPa for F3 (se: 68.6%, sp: 71.0%), and 11.6 kPa for F4 (se: 76.9%, sp: 84.9%) (Table 3). The AUCs of LSM-VCTE for diagnosing fibrosis stages F2, F3, and F4 were 0.74, 0.81, and 0.92, respectively, all comparable to the diagnostic performance of LSM-SWE (Fig. S2, Table S3).

**Table 3:** Diagnostic performance of 2D-SWE for liver fibrosis

Fibrosis stage	Cut-off (kPa)	Se (%)	Sp (%)	NPV (%)	PPV (%)	AUC
F2	8.7	67.3	76.5	44.8	89.2	0.73
F3	9.1	68.6	71.0	66.7	72.7	0.79
F4	11.6	76.9	84.9	93.8	55.6	0.88

SWE shear wave elastography, Se sensitivity, Sp specificity, NPV negative predictive value, PPV positive predictive value, AUC area under the receiver-operating characteristics curve

### Relationship between ATI and CAP and associated factors

**Fig. 2:**
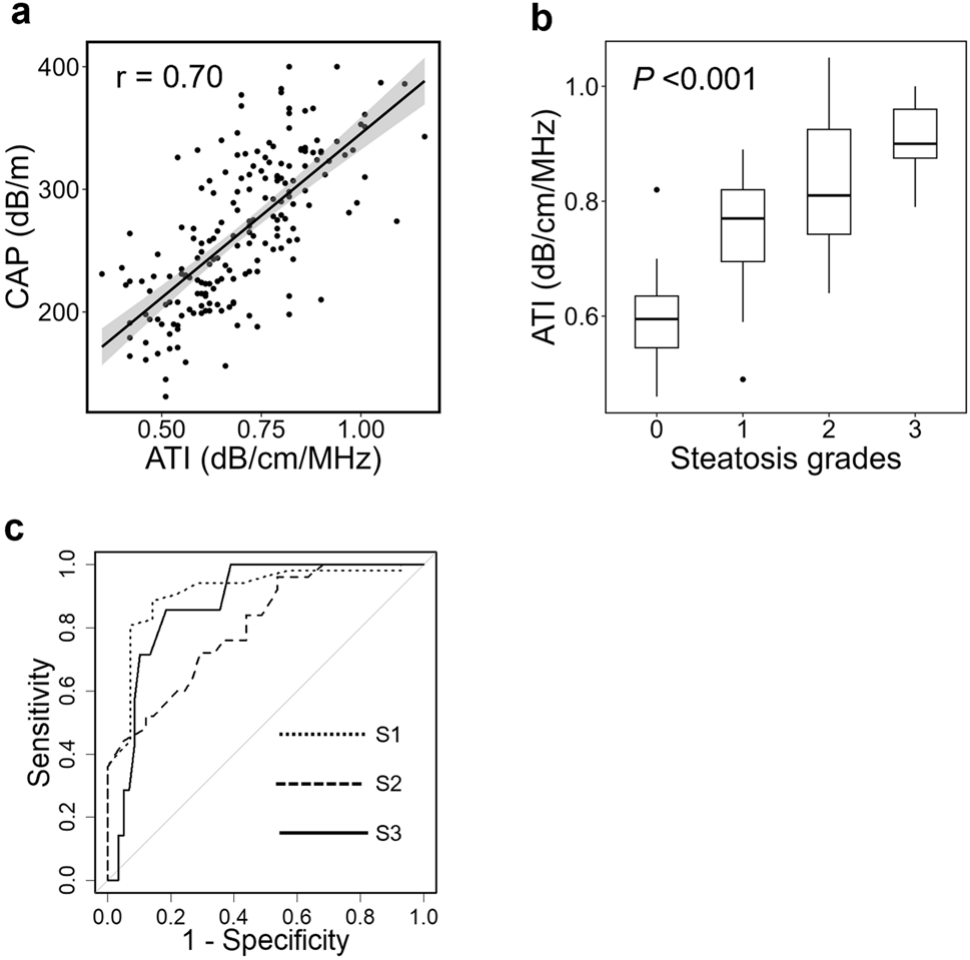
Diagnostic performance of ATI in patients with chronic liver disease. **a** ATI and CAP showed a positive correlation (r = 0.70, *P* < 0.001, Pearson's product-rate correlation coefficient analysis). **b** ATI increased significantly according to hepatic steatosis grade (*P* < 0.001, Jonckheere-Terpstra trend test). **c** Receiver operating characteristic curves of ATI to diagnose hepatic steatosis grades. ATI: attenuation imaging, CAP: controlled attenuation parameter

ATI and CAP showed a strong positive correlation (r = 0.70, *P* < 0.001) (Fig. 2a). Univariate analysis revealed that BMI, age, male sex, platelet count, AST, ALT, GGT, and SCD levels were significantly associated with ATI. All these factors were associated with ATI, except for GGT. The multivariate analysis revealed that BMI was an independent factor associated with ATI and CAP, whereas SCD was significantly associated only with CAP (Table 4).

**Table 4:** Factors associated with ATI and CAP

Variable	ATI				CAP			
	Univariate		Multivariate		Univariate		Multivariate	
	β	*P* value	β	*P* value	β	*P* value	β	*P* value
BMI	0.50	** < 0.001**	0.48	** < 0.001**	0.59	** < 0.001**	0.34	**0.004**
Age	– 0.20	**0.01**	0.00	> 0.9	– 0.28	** < 0.001**	– 0.03	0.70
Male	0.18	**0.01**	– 0.02	0.80	0.28	** < 0.001**	0.06	0.40
Platelet count	0.17	**0.02**	0.11	0.13	0.19	**0.01**	0.09	0.20
Albumin	0.07	0.30			0.10	0.20		
AST	0.20	**0.01**			0.19	**0.01**		
ALT	0.23	**0.001**	– 0.02	0.80	0.28	** < 0.001**	0.00	> 0.9
GGT	0.22	**0.002**	0.11	0.094	0.07	0.30		
Total bilirubin	0.05	0.50			0.02	0.70		
SCD	0.51	** < 0.001**	0.00	> 0.9	0.67	** < 0.001**	0.26	**0.03**

ATI attenuation imaging, CAP controlled attenuation parameter, BMI body mass index, AST asparagine aminotransferase, ALT alanine aminotransferase, GGT γ-glutamyltransferase, SCD skin-capsular distance

ATI measurements obtained with the Aplio a550 and Aplio i700 were comparable (Table S2). Furthermore, the correlation between ATI and CAP was equally strong whether using the Aplio a550 (r = 0.59, *P* < 0.001) or the Aplio i700 (r = 0.75, *P* < 0.001) (Fig. S3).

### Diagnostic ability of ATI for hepatic steatosis

In cases where liver biopsy was performed, ATI measurements increased significantly according to the hepatic steatosis grade (Fig. 2b). The AUCs of ATI for the diagnosis of hepatic steatosis grades S1, S2, and S3 were 0.91, 0.80, and 0.88, respectively (Fig. 2c). The optimal cut-off levels of ATI for diagnosing hepatic steatosis were 0.66 dB/cm/MHz for S1 (se: 88.5%, sp: 85.7%), 0.79 dB/cm/MHz for S2 (se: 72.0%, sp: 70.7%), and 0.86 dB/cm/MHz for S3 (se: 85.7%, sp: 81.4%) (Table 5). The AUCs of CAP for diagnosing steatosis grades S1, S2, and S3 were, 0.91, 0.80, and 0.80, respectively, all comparable to the diagnostic performance of ATI (Fig. S4, Table S4).

**Table 5:** Diagnostic performance of ATI for hepatic steatosis

Steatosis grade	Cut-off (dB/cm/MHz)	Se (%)	Sp (%)	NPV (%)	PPV (%)	AUC
S1	0.66	88.5	85.7	66.7	95.8	0.91
S2	0.79	72.0	70.7	80.6	60.0	0.80
S3	0.86	85.7	81.4	98.0	35.3	0.88

ATI attenuation imaging, Se sensitivity, Sp specificity, NPV negative predictive value, PPV positive predictive value, AUC area under the receiver-operating characteristics curve

### Diagnostic performance of LSM-SWE and ATI in patients with MASLD

**Fig. 3:**
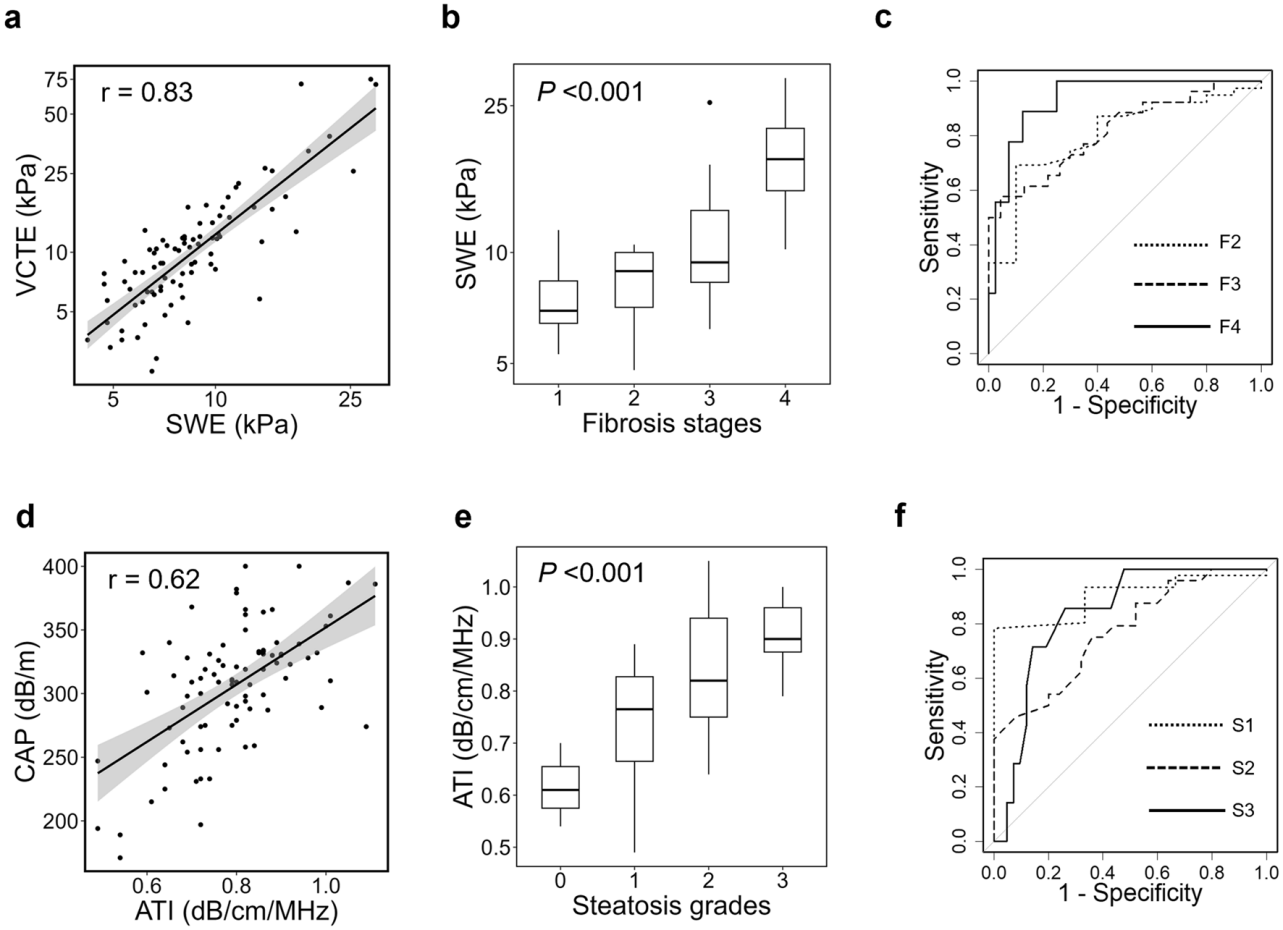
Diagnostic performance of 2D-SWE and ATI in patients with MASLD. **a** 2D-SWE and VCTE showed a positive correlation (r = 0.83, *P* < 0.001, Pearson product-rate correlation coefficient analysis). **b** LSM using 2D-SWE significantly increased according to the liver fibrosis stage (*P* < 0.001, Jonckheere-Terpstra trend test). **c** Receiver operating characteristic curves of 2D-SWE for diagnosing the stages of liver fibrosis. **d** ATI and CAP levels were positively correlated (r = 0.62, *P* < 0.001, Pearson product-rate correlation coefficient analysis). **e** ATI increased significantly, according to the hepatic steatosis grade (*P* < 0.001, Jonckheere-Terpstra trend test). **f** The receiver operating characteristic curves of ATI used to diagnose hepatic steatosis grades. 2D-SWE: two-dimensional shear-wave elastography, ATI: attenuation imaging, VCTE: vibration-controlled transient elastography, CAP: controlled attenuation parameter

A subgroup analysis was performed in patients with MASLD. The patient characteristics are shown in Table S5. LSM-SWE and LSM-VCTE showed very strong positive correlations in patients with MASLD (r = 0.83, *P* < 0.001) (Fig. 3a). In cases where a liver biopsy was performed, LSM-SWE measurements increased significantly according to the liver fibrosis stage (Fig. 3b). The AUCs of LSM-SWE for diagnosing F2, F3, and F4 were 0.80, 0.82, and 0.93, respectively (Fig. 3c, Table S6). Multivariate analysis revealed that platelet count and GGT were independently associated with LSM-SWE and LSM-VCTE in patients with MASLD, which is consistent with the results of the analysis in the full population (Table S7).

The ATI and CAP levels showed a strong positive correlation in patients with MASLD (r = 0.62, *P* < 0.001) (Fig. 3d). In cases where liver biopsy was performed, ATI measurements increased significantly according to the hepatic steatosis grade (Fig. 3e). The AUCs of ATI for diagnosing S1, S2, and S3 were 0.90, 0.77, and 0.84, respectively (Fig. 3f, Table S8). Univariate analysis revealed that BMI, AST and ALT levels, and SCD were significantly associated with the ATI and CAP levels. Multivariate analysis revealed that ALT was an independent factor associated with ATI and CAP, whereas BMI was significantly associated only with ATI in patients with MASLD (Table S9).

## Discussion

In this study, we investigated the diagnostic performance of 2D-SWE and ATI using the Canon US system for liver fibrosis and steatosis in patients with CLD of various etiologies. 2D-SWE and ATI were strongly correlated with VCTE and CAP using FibroScan®, respectively, a device with much evidence in the diagnosis of liver fibrosis and steatosis [5, 14]. Furthermore, 2D-SWE and ATI exhibited good diagnostic performance in identifying histological fibrosis and steatosis grades, respectively. In recent years, there has been a trend towards using noninvasive LSM-VCTE instead of invasive liver biopsies to stratify the risk of liver-related events in advanced CLD [23]. However, FibroScan® is a special, expensive device and is constrained by being usable only in specialized hospitals. In contrast, 2D-SWE and ATI can be measured with just an ultrasound device, and they offer the stability of measurement values because the region of interest can be determined in real time while viewing the B-mode screen. Our insights further enhance the value of 2D-SWE and ATI as widely used, highly versatile, and potential alternatives to FibroScan®.

Regarding liver fibrosis assessment, we found a strong positive correlation between LSM using 2D-SWE and VCTE, indicating that 2D-SWE has sufficient potential as a substitute for VCTE. This result is in line with previous reports demonstrating that 2D-SWE using the Canon US system correlates well with VCTE [18, 24–27]. Platelet count and GGT were independent factors associated with LSM using 2D-SWE and VCTE, with no marked differences in factors affecting the test values, thereby supporting the similar characteristics of both tests. Importantly, the Bland–Altman analysis revealed a proportional error between LSM using 2D-SWE and VCTE. The higher the LSM value was, the greater the difference between the measured values became. LSM using 2D-SWE tended to be smaller than that using VCTE. A previous report consistently demonstrated that LSM using 2D-SWE tended to be lower than that using VCTE when the LSM using VCTE was higher than 12 kPa [24]. Therefore, caution should be exercised when estimating liver fibrosis using 2D-SWE, especially in cases with high LSM values. This is because assessing LSM using 2D-SWE at the same cut-off value as LSM using VCTE may underestimate liver fibrosis. This observation underlines the need to establish specific cut-off values for the diagnosis of liver fibrosis using 2D-SWE.

Our results demonstrated that LSM using 2D-SWE exhibited good diagnostic performance in identifying fibrosis stages. Studies have compared LSM using 2D-SWE on the Canon US system and histological liver fibrosis stage. Uchikawa et al. reported that in 85 patients with CLD of various etiologies, AUCs for the diagnostic performance of F1, F2, and F3 were 0.652, 0.732, and 0.761, respectively, which were not significantly different from those of LSM using VCTE [28]. Iijima et al. reported that in 109 patients with CLD of various etiologies, AUCs for the diagnostic performance of F2 and F4 were 0.855 and 0.967, respectively [27]. These findings, including ours, support the potential of 2D-SWE as a reliable noninvasive tool for liver fibrosis staging in patients with CLD. In other reports on patients with biopsy-proven NAFLD, AUCs for predicting each fibrosis stage based on 2D-SWE using the Canon US system were > 0.8, which is comparable to our results [29, 30]. It is suggested that the diagnostic performance of 2D-SWE for liver fibrosis may be even better if appropriate cut-off values are set for each cause of CLD.

Regarding hepatic steatosis assessment, we found a strong positive correlation between ATI and CAP, indicating that ATI can provide a reliable measurement of ultrasound attenuation associated with hepatic steatosis. This result is consistent with previous reports demonstrating that ATI correlates well with CAP [18, 25, 26]. The multivariate analysis revealed that only BMI was an independent factor associated with ATI and CAP, highlighting its influence on these measurements. Importantly, SCD, a known factor limiting the accuracy of elastography, is associated with CAP but not ATI. It has been suggested that ATI can be used to assess hepatic steatosis, regardless of SCD, which is advantageous over CAP. We observed that ATI exhibited good diagnostic performance in identifying steatosis grades. Our results are consistent with previous reports demonstrating AUCs of 0.81–0.93, 0.86–0.91, and 0.79–0.93 for S1, S2, and S3 detection, respectively [15, 29–32]. These findings suggest that ATI could serve as a valuable tool for assessing hepatic steatosis in patients with CLD.

In the subgroup analysis specifically focusing on patients with MASLD, LSM using 2D-SWE and ATI was strongly correlated with VCTE and CAP, respectively, comparable to the study in all patients with CLD. Obesity, particularly high SCD, is a limiting factor for the accuracy of elastography, and previous reports have demonstrated a lower accuracy rate for elastography in a group with SCD > 22.5 mm [28, 33]. However, our multivariate analysis showed that SCD was not an independent factor affecting 2D-SWE findings in patients with MASLD. Moreover, as previously reported [15], the multivariate analysis revealed that SCD was not an independent factor associated with ATI. These findings indicate that both techniques are effective for evaluating liver fibrosis and steatosis in patients with MASLD.

This study had several limitations that should be considered. The study was conducted at a single institution with a relatively small sample size, which limits the generalizability of the findings. Therefore, there was limited discussion on the diagnostic performance in diverse populations, including different racial/ethnic groups, geographical regions, sexes, and age groups. Furthermore, the study population primarily consisted of patients who agreed to undergo liver biopsy, resulting in a potential selection bias. Additionally, 2D-SWE and ATI measurements are device dependent, and further validation across different devices and settings is required. Future research should address these limitations and validate the diagnostic performance of 2D-SWE and ATI in different populations and CLD etiologies.

## Conclusion

Our study provides evidence supporting the diagnostic performance of 2D-SWE and ATI in the assessment of liver fibrosis and steatosis in patients with CLD. Both techniques demonstrated good accuracy and strong correlations with the reference standards (LSM and CAP using VCTE). These noninvasive imaging techniques hold promise as valuable tools for CLD evaluation and management, offering advantages over invasive procedures, such as liver biopsy. However, further research and validation in larger cohorts are necessary to establish their widespread clinical utility and integration into routine practice for CLD diagnosis and monitoring.

## Supplementary Information

Below is the link to the electronic supplementary material.

Supplementary file1 (TIF 312 KB)

Supplementary file2 (TIF 519 KB)

Supplementary file3 (TIF 341 KB)

Supplementary file4 (TIF 516 KB)

Supplementary file5 (DOCX 16 KB)

Supplementary file6 (DOCX 40 KB)

## Data Availability

The authors confirm that the data supporting the findings of this study are available within the article and its supplementary materials.

## Abbreviations

2D-SWE: Two-dimensional shear wave elastography
ALD: Alcohol-related liver disease
ALT: Alanine aminotransferase
AST: Asparagine aminotransferase
ATI: Attenuation imaging
AUC: Area under the receiver-operating characteristics curve
BMI: Body mass index
CAP: Controlled attenuation parameter
CLD: Chronic liver diseases
GGT: γ-Glutamyltransferase
IQR: Interquartile range
LSM: Liver stiffness measurement
MASLD: Metabolic dysfunction-associated fatty liver disease
NAFLD: Non-alcoholic fatty liver disease
SCD: Skin-capsular distance
VCTE: Vibration-controlled transient elastography
