# Supplementary figures and images for: Diagnostic performance of two-dimensional shear wave elastography and attenuation imaging for fibrosis and steatosis assessment in chronic liver disease
Source: J Med Ultrason (2001). 2024 Jun 29;52(1):95–103. doi: 10.1007/s10396-024-01473-5 (PMC11799025; doi:10.1007/s10396-024-01473-5)

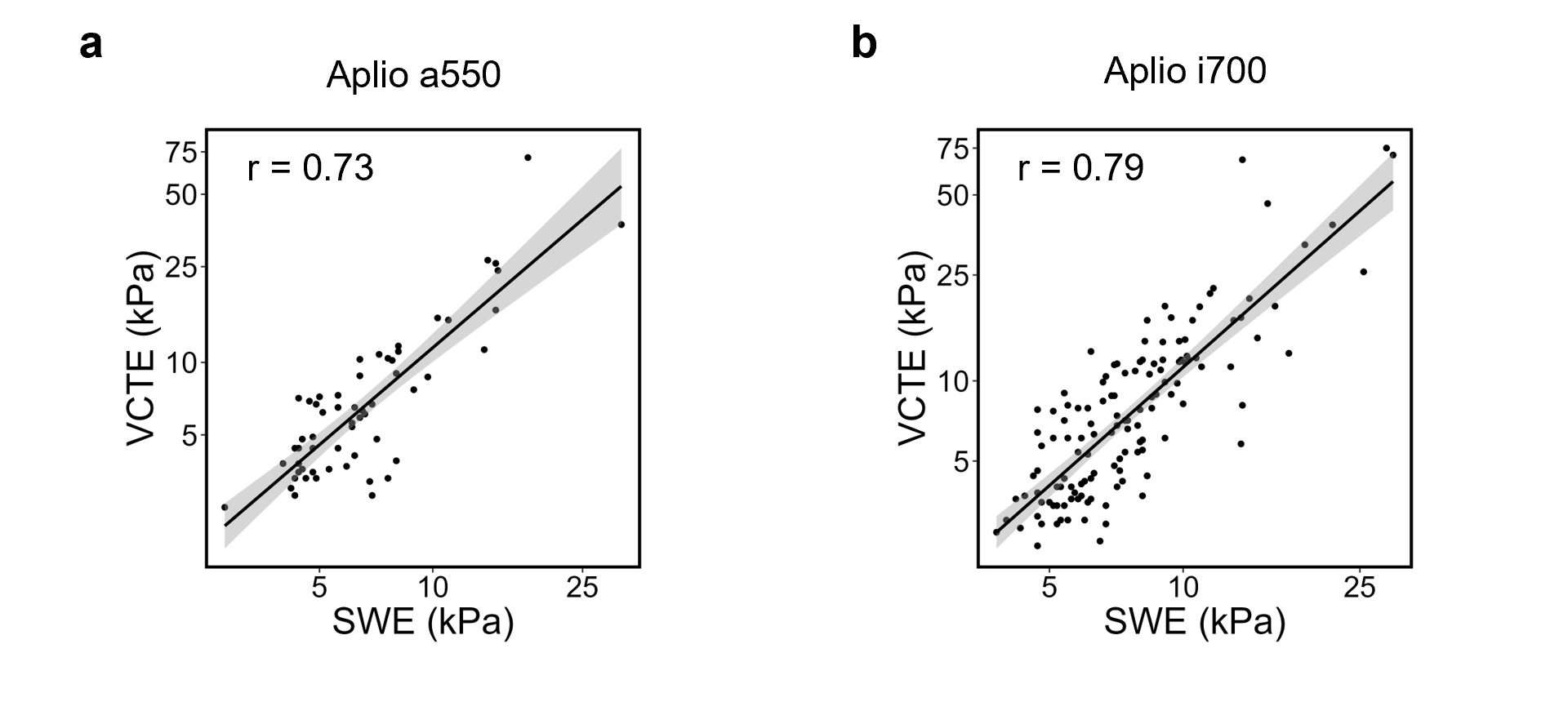

Supplement: Supplementary file 1 — Supplementary file1 (TIF 312 KB) [file 10396_2024_1473_MOESM1_ESM.tif]

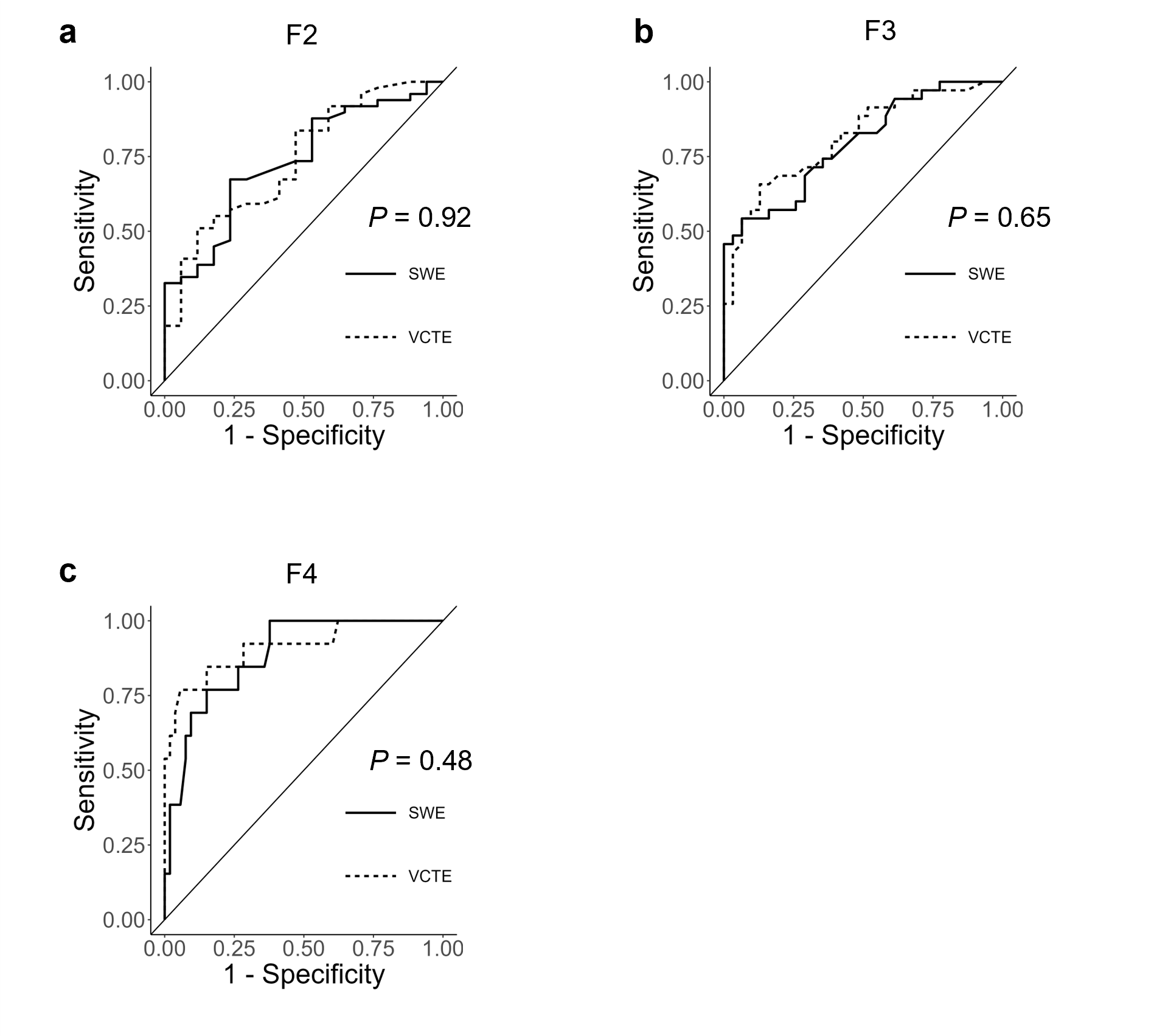

Supplement: Supplementary file 2 — Supplementary file2 (TIF 519 KB) [file 10396_2024_1473_MOESM2_ESM.tif]

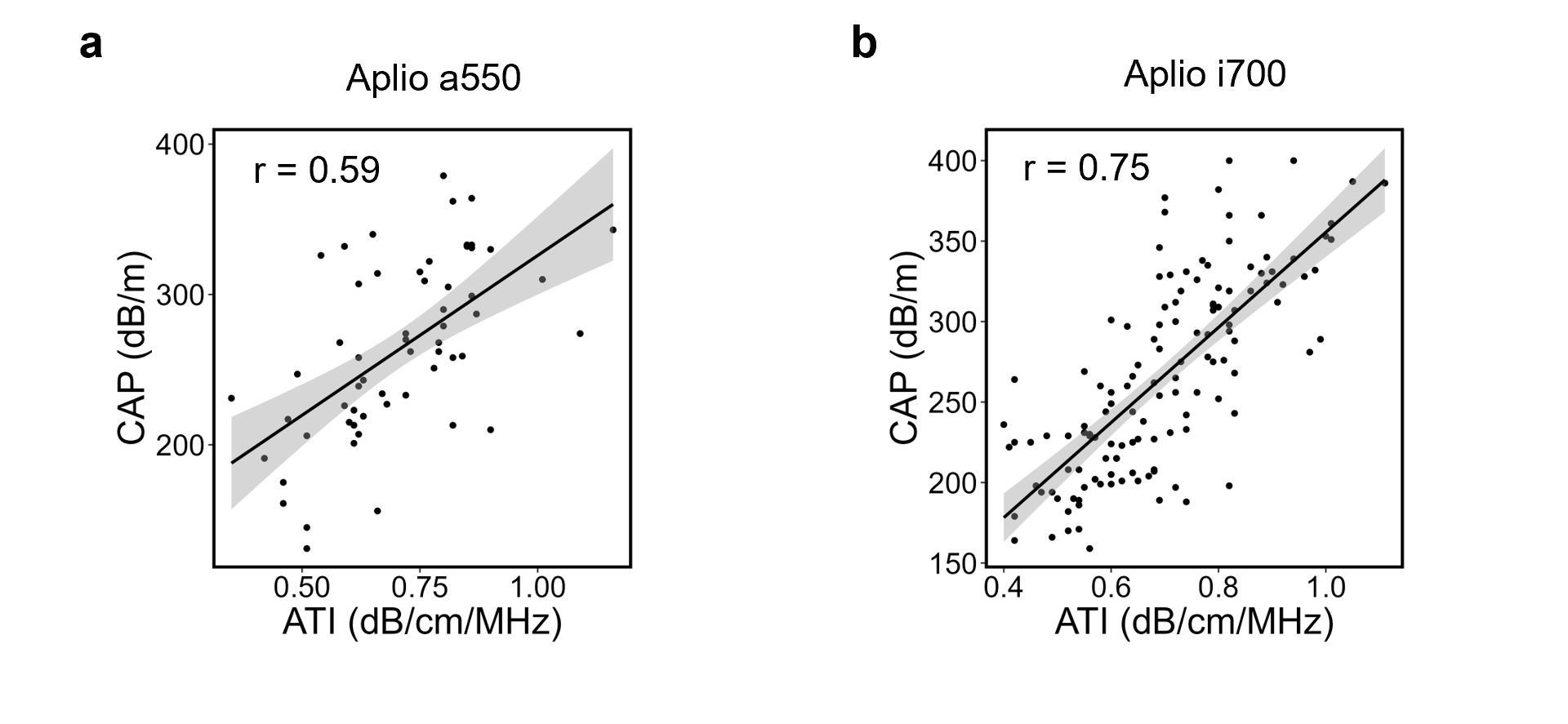

Supplement: Supplementary file 3 — Supplementary file3 (TIF 341 KB) [file 10396_2024_1473_MOESM3_ESM.tif]

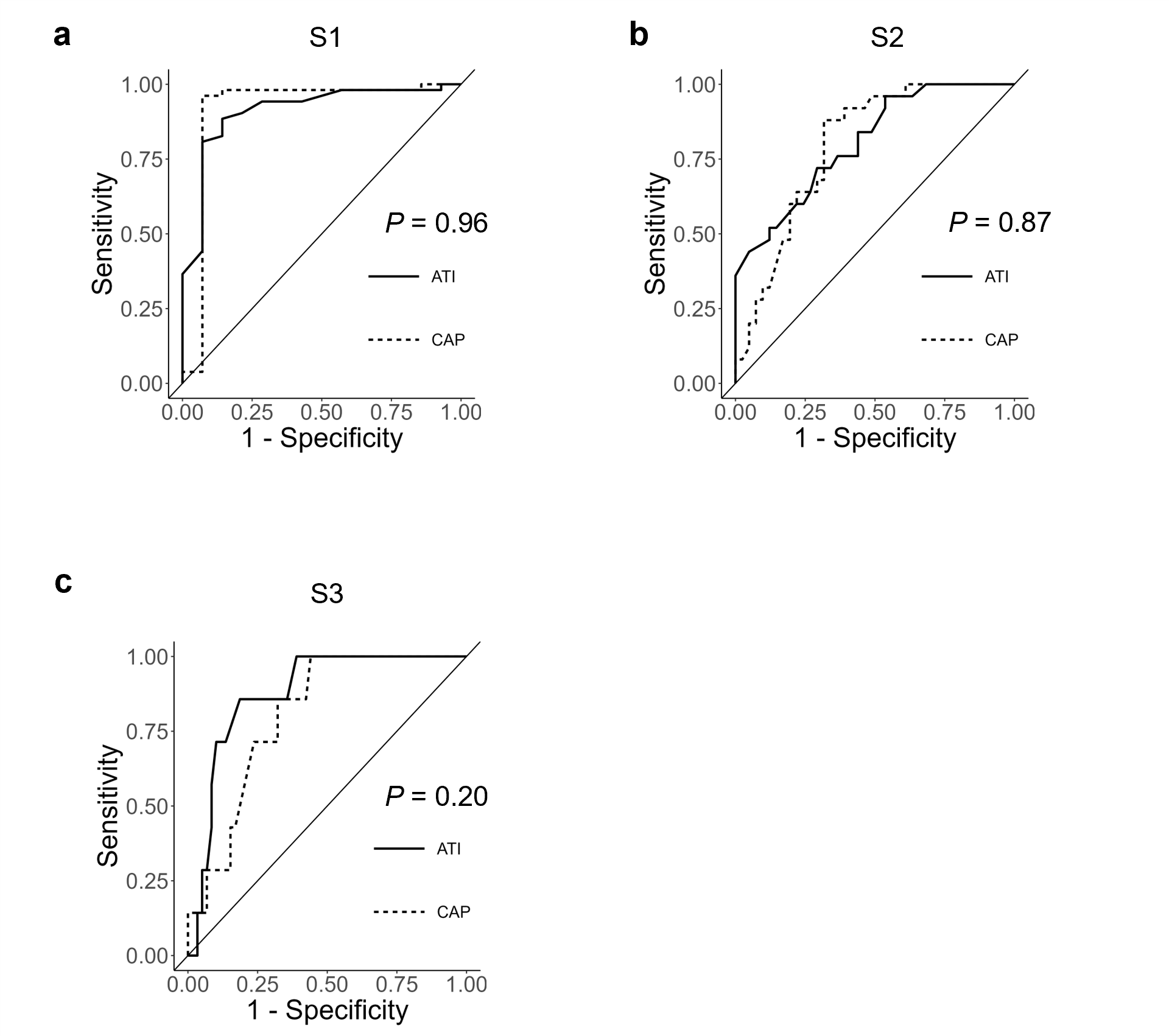

Supplement: Supplementary file 4 — Supplementary file4 (TIF 516 KB) [file 10396_2024_1473_MOESM4_ESM.tif]
