# Supplementary material for: Diagnostic performance of two-dimensional shear wave elastography and attenuation imaging for fibrosis and steatosis assessment in chronic liver disease
Source: J Med Ultrason (2001). 2024 Jun 29;52(1):95–103. doi: 10.1007/s10396-024-01473-5 (PMC11799025; doi:10.1007/s10396-024-01473-5)
Supplement: Supplementary file 5 — Supplementary file5 (DOCX 16 KB) [file 10396_2024_1473_MOESM5_ESM.docx]

**Fig S1. Correlation of 2D-SWE and VCTE per device**

2D-SWE and VCTE were positively correlated in both (a) Aplio a550 (N = 58) and (b) Aplio i700 (N = 132) (r = 0.73 and r = 0.79, respectively, Pearson product-rate correlation coefficient analysis).

**Fig S2. Comparison of diagnostic performance of 2D-SWE and VCTE**

Receiver operating characteristic curves of 2D-SWE and VCTE for diagnosing each stage of liver fibrosis. Diagnostic performance for (a) F2, (b) F3, and (c) F4 was comparable between 2D-SWE and VCTE (*P* = 0.92, *P* = 0.65, and *P* = 0.48, respectively, DeLong’s test).

**Fig S3. Correlation of ATI and CAP per device**

ATI and CAP were positively correlated in both (a) Aplio a550 (N = 58) and (b) Aplio i700 (N = 132) (r = 0.59 and r = 0.75, respectively, Pearson product-rate correlation coefficient analysis).

**Fig S4. Comparison of diagnostic performance of ATI and CAP**

Receiver operating characteristic curves of ATI and CAP for diagnosing each grade of hepatic steatosis. Diagnostic performance for (a) S1, (b) S2, and (c) S3 was comparable between ATI and CAP (*P* = 0.96, *P* = 0.87, and *P* = 0.20, respectively, DeLong’s test).
