# Supplementary material for: Diagnostic performance of two-dimensional shear wave elastography and attenuation imaging for fibrosis and steatosis assessment in chronic liver disease
Source: J Med Ultrason (2001). 2024 Jun 29;52(1):95–103. doi: 10.1007/s10396-024-01473-5 (PMC11799025; doi:10.1007/s10396-024-01473-5)
Supplement: Supplementary file 6 — Supplementary file6 (DOCX 40 KB) [file 10396_2024_1473_MOESM6_ESM.docx]

**Table S1. Etiologies of CLD in liver biopsy cases**

| **Etiology** | **Patients with CLD (n = 66)** | |
| --- | --- | --- |
| HCV | 4 | (6.1) |
| MASLD | 49 | (74.2) |
| ALD | 4 | (6.1) |
| AIH | 6 | (9.1) |
| PBC | 2 | (3.0) |
| Cryptogenic | 1 | (1.5) |

Data are expressed as number (percentages). CLD, chronic liver disease; HCV, hepatitis C virus; SVR, sustained virological response; MASLD, metabolic dysfunction-associated steatotic liver disease; ALD, alcohol-related liver disease; AIH. autoimmune hepatitis; PBC, primary biliary cholangitis.

**Table S2. Patient characteristics divided by ultrasound equipment**

| **Variable** | **Aplio i700 (n = 132)** | | **Aplio a550 (n = 58)** | | ***P* value** |
| --- | --- | --- | --- | --- | --- |
| BMI | 25.7 | (22.8–29.6) | 28.2 | (24.3–30.7) | 0.12 |
| Age | 66 | (52–74) | 61 | (53–73) | 0.47 |
| Male, % | 67 | (51) | 30 | (53) | 0.81 |
| Platelet count, ×10^3^/μL | 218 | (178–265) | 217 | (187–255) | 0.54 |
| Albumin, g/dL | 4.3 | (4.1–4.5) | 4.3 | (4.2–4.6) | 0.16 |
| AST, U/L | 31 | (23–56) | 29 | (20–40) | 0.08 |
| ALT, U/L | 30 | (17–57) | 25 | (16–45) | 0.07 |
| GGT, U/L | 43 | (25–89) | 32 | (19–88) | 0.45 |
| Total bilirubin, mg/dL | 0.8 | (0.6–1) | 0.9 | (0.7–1.3) | **0.02** |
| FIB-4 index | 1.67 | (1.12–2.58) | 1.49 | (1.27–1.91) | 0.15 |
| SCD, cm | 1.79 | (1.46–2.07) | 1.75 | (1.43–2.07) | 0.53 |
| LSM-VCTE, kPa | 7.1 | (4.2–11.7) | 6.2 | (3.8–9.9) | 0.48 |
| LSM-SWE, kPa | 7.2 | (5.7–9.2) | 6.2 | (4.8–8.0) | 0.20 |
| CAP, dB/m | 261 | (215–314) | 262 | (220–313) | 0.83 |
| ATI, dB/cm/MHz | 0.69 | (0.58–0.80) | 0.72 | (0.61–0.82) | 0.66 |
| Fibrosis stage |  |  |  |  |  |
| 0-1 | 15 | (25.9) | 2 | (25.0) |  |
| 2 | 14 | (24.1) | 0 | (0.0) |  |
| 3 | 19 | (32.8) | 3 | (37.5) |  |
| 4 | 10 | (17.2) | 3 | (37.5) |  |
| Steatosis grade |  |  |  |  |  |
| 0 | 14 | (24.1) | 0 | (0.0) |  |
| 1 | 22 | (37.9) | 5 | (62.5) |  |
| 2 | 15 | (25.9) | 3 | (37.5) |  |
| 3 | 7 | (12.1) | 0 | (0.0) |  |
| Etiology |  |  |  |  |  |
| HBV | 15 | (11.4) | 13 | (22.4) |  |
| HCV (SVR) | 26 | (19.7) | 13 | (22.4) |  |
| MASLD | 61 | (46.2) | 25 | (43.1) |  |
| ALD | 6 | (4.5) | 3 | (5.2) |  |
| AIH | 12 | (9.1) | 2 | (3.5) |  |
| PBC | 9 | (6.8) | 2 | (3.5) |  |
| Cryptogenic | 3 | (2.3) | 0 | (0.0) |  |

Data are expressed as the median (25th–75th percentiles) or number (percentages). BMI, body mass index; AST, asparagine aminotransferase; ALT alanine aminotransferase; GGT, γ-glutamyltransferase; FIB-4, fibrosis 4; SCD, skin-capsular distance; LSM, liver stiffness measurement; VCTE, vibration-controlled transient elastograhpy; SWE, shear wave elastography; CAP, controlled attenuation parameter; ATI, Attenuation Imaging; HBV, hepatitis B virus; HCV, hepatitis C virus; SVR, sustained virological response; MASLD, metabolic dysfunction-associated steatotic liver disease; ALD, alcohol-related liver disease; AIH. autoimmune hepatitis; PBC, primary biliary cholangitis.

**Table S3. Diagnostic performance of VCTE for liver fibrosis**

| **Fibrosis stage** | **Se (%)** | **Sp (%)** | **NPV (%)** | **PPV (%)** | **AUC** |
| --- | --- | --- | --- | --- | --- |
| F2 | 55.1 | 82.4 | 38.9 | 90.0 | 0.74 |
| F3 | 65.7 | 87.1 | 69.2 | 85.2 | 0.81 |
| F4 | 84.6 | 84.9 | 95.7 | 57.9 | 0.92 |

VCTE, vibration-controlled transient elastograhpy; Se, Sensitivity; Sp, Specificity; NPV, negative predictive value; PPV, positive predictive value; AUC, area under the receiver-operating characteristics curve.

**Table S4. Diagnostic performance of CAP for hepatic steatosis**

| **Steatosis grade** | **Se (%)** | **Sp (%)** | **NPV (%)** | **PPV (%)** | **AUC** |
| --- | --- | --- | --- | --- | --- |
| S1 | 96.2 | 92.9 | 86.7 | 98.0 | 0.91 |
| S2 | 88.0 | 68.3 | 90.3 | 62.9 | 0.80 |
| S3 | 85.7 | 67.8 | 97.6 | 24.0 | 0.80 |

CAP, controlled attenuation parameter; Se, Sensitivity; Sp, Specificity; NPV, negative predictive value; PPV, positive predictive value; AUC, area under the receiver-operating characteristics curve.

**Table S5. Patient characteristics in MASLD group**

| **Variable** | **MASLD group (n = 86)** | |
| --- | --- | --- |
| BMI | 28.2 | (24.9–31.4) |
| Age | 58 | (47–72) |
| Male, % | 55 | (64) |
| Platelet count, ×10^3^/μL | 230 | (189–280) |
| Albumin, g/dL | 4.4 | (4.1–4.5) |
| AST, U/L | 42 | (30–70) |
| ALT, U/L | 50 | (30–94) |
| GGT, U/L | 63 | (43–103) |
| Total bilirubin, mg/dL | 0.9 | (0.7–1.1) |
| FIB-4 index | 1.58 | (1.12–2.16) |
| SCD, cm | 2.04 | (1.79–2.18) |
| LSM-VCTE, kPa | 9.9 | (6.4–12.9) |
| LSM-SWE, kPa | 8.0 | (6.5–10.2) |
| CAP, dB/m | 312 | (276–332) |
| ATI, dB/cm/MHz | 0.80 | (0.72–0.86) |
| Fibrosis stage 0-1/2/3/4 |  |  |
| 0-1 | 10 | (20.4) |
| 2 | 13 | (26.5) |
| 3 | 17 | (34.7) |
| 4 | 9 | (18.4) |
| Steatosis grade |  |  |
| 0 | 3 | (6.1) |
| 1 | 22 | (44.9) |
| 2 | 17 | (34.7) |
| 3 | 7 | (14.3) |

Data are expressed as the median (25th–75th percentiles) or number (percentages).

MASLD, metabolic dysfunction-associated steatotic liver disease; BMI, body mass index; AST, asparagine aminotransferase; ALT alanine aminotransferase; GGT, γ-glutamyltransferase; FIB-4, fibrosis 4; SCD, skin-capsular distance; LSM, liver stiffness measurement; VCTE, vibration-controlled transient elastograhpy; SWE, shear-wave elastography; CAP, controlled attenuation parameter; ATI, attenuation imaging.

**Table S6. Diagnostic performance of 2D-SWE for liver fibrosis in biopsy-proven MASLD**

| **Fibrosis stage** | **Cut-off (kPa)** | **Se (%)** | **Sp (%)** | **NPV (%)** | **PPV (%)** | **AUC** |
| --- | --- | --- | --- | --- | --- | --- |
| F2 | 8.7 | 69.2 | 90.0 | 42.9 | 96.4 | 0.80 |
| F3 | 9.2 | 69.2 | 73.9 | 68.0 | 75.0 | 0.82 |
| F4 | 11.6 | 88.9 | 87.5 | 97.2 | 61.5 | 0.93 |

SWE, shear-wave elastography; MASLD, metabolic dysfunction-associated steatotic liver disease; Se, Sensitivity; Sp, Specificity; NPV, negative predictive value; PPV, positive predictive value; AUC, area under the receiver-operating characteristics curve.

**Table S7. Factors associated with 2D-SWE and VCTE in patients with MASLD**

|  | **2D-SWE** | | | | |  | **VCTE** | | | | |
| --- | --- | --- | --- | --- | --- | --- | --- | --- | --- | --- | --- |
|  | **Univariate** | |  | **Multivariable** | |  | **Univariate** | |  | **Multivariable** | |
| **variable** | **β** | ***P* value** |  | **β** | ***P* value** |  | **β** | ***P* value** |  | **β** | ***P* value** |
| BMI | 0.25 | **0.04** |  | 0.17 | 0.12 |  | 0.29 | **0.01** |  | 0.19 | **0.04** |
| Age | 0.05 | 0.60 |  |  |  |  | 0.11 | 0.30 |  |  |  |
| Male | 0.13 | 0.20 |  |  |  |  | 0.10 | 0.40 |  |  |  |
| Platelet count | -0.20 | 0.06 |  | -0.33 | **0.002** |  | -0.29 | **0.01** |  | -0.39 | **<0.001** |
| Albumin | -0.16 | 0.20 |  |  |  |  | -0.25 | **0.02** |  | -0.15 | 0.12 |
| AST | 0.25 | **0.02** |  | 0.02 | 0.90 |  | 0.30 | **0.004** |  | 0.01 | 0.90 |
| ALT | 0.07 | 0.50 |  |  |  |  | 0.12 | 0.30 |  |  |  |
| GGT | 0.43 | **<0.001** |  | 0.44 | **<0.001** |  | 0.48 | **<0.001** |  | 0.52 | **<0.001** |
| Total bilirubin | 0.19 | 0.09 |  |  |  |  | 0.14 | 0.20 |  |  |  |
| SCD | 0.17 | 0.12 |  |  |  |  | 0.21 | 0.06 |  |  |  |

SWE, shear-wave elastography; VCTE, vibration-controlled transient elastograhpy; MASLD, metabolic dysfunction-associated steatotic liver disease; BMI, body mass index; AST, asparagine aminotransferase; ALT alanine aminotransferase; GGT, γ-glutamyltransferase; SCD, skin-capsular distance.

**Table S8. Diagnostic performance of ATI for hepatic steatosis in biopsy-proven MASLD**

| **Steatosis grade** | **Cut-off (dB/cm/MHz)** | **Se (%)** | **Sp (%)** | **NPV (%)** | **PPV (%)** | **AUC** |
| --- | --- | --- | --- | --- | --- | --- |
| S1 | 0.71 | 78.3 | 100 | 23.1 | 100 | 0.90 |
| S2 | 0.79 | 75.0 | 64.0 | 72.7 | 66.7 | 0.77 |
| S3 | 0.86 | 85.7 | 73.8 | 96.9 | 35.3 | 0.84 |

ATI, attenuation imaging; MASLD, metabolic dysfunction-associated steatotic liver disease; Se, Sensitivity; Sp, Specificity; NPV, negative predictive value; PPV, positive predictive value; AUC, area under the receiver-operating characteristics curve.

**Table S9. Factors associated with ATI and CAP in patients with MASLD**

|  | **ATI** | | | | |  | **CAP** | | | | |
| --- | --- | --- | --- | --- | --- | --- | --- | --- | --- | --- | --- |
|  | **Univariate** | |  | **Multivariable** | |  | **Univariate** | |  | **Multivariable** | |
| **variable** | **β** | ***P* value** |  | **β** | ***P* value** |  | **β** | ***P* value** |  | **β** | ***P* value** |
| BMI | 0.32 | **0.004** |  | 0.39 | **0.01** |  | 0.38 | **<0.001** |  | 0.24 | 0.09 |
| Age | -0.15 | 0.20 |  |  |  |  | -0.20 | 0.06 |  |  |  |
| Male | 0.05 | 0.70 |  |  |  |  | 0.19 | 0.08 |  |  |  |
| Platelet count | 0.10 | 0.40 |  |  |  |  | 0.04 | 0.70 |  |  |  |
| Albumin | 0.14 | 0.20 |  |  |  |  | 0.11 | 0.30 |  |  |  |
| AST | 0.22 | **0.04** |  |  |  |  | 0.24 | **0.02** |  |  |  |
| ALT | 0.28 | **0.01** |  | 0.23 | **0.04** |  | 0.34 | **0.002** |  | 0.20 | **0.047** |
| GGT | 0.18 | 0.10 |  |  |  |  | 0.13 | 0.20 |  |  |  |
| Total bilirubin | -0.13 | 0.20 |  |  |  |  | -0.11 | 0.30 |  |  |  |
| SCD | 0.24 | **0.02** |  | -0.19 | 0.20 |  | 0.42 | **<0.001** |  | 0.12 | 0.40 |

ATI, attenuation imaging; CAP, controled attenuation parameter; MASLD, metabolic dysfunction-associated steatotic liver disease; BMI, body mass index; AST, asparagine aminotransferase; ALT alanine aminotransferase; GGT, γ-glutamyltransferase; SCD, skin-capsular distance; LSM, liver stiffness measurement.
